# Upper respiratory tract detection of *Mycoplasma ovipneumoniae* employing nasopharyngeal swabs

**DOI:** 10.1186/s12917-024-04342-y

**Published:** 2024-11-01

**Authors:** David R. Herndon, Paige C. Grossman, Julianne K. Hwang, Lindsay M.W. Piel

**Affiliations:** 1 USDA-ARS Animal Disease Research Unit, Pullman, WA 99164 USA; 2 Department of Veterinary Microbiology and Pathology, Washington State University, Pullman, WA 99164 USA

**Keywords:** *Mycoplasma ovipneumoniae*, Nasopharyngeal, Nasal swab, Inhibition, Quantitative PCR, Diagnostic test

## Abstract

**Background:**

Flock-level prevalence and characterization of *Mycoplasma ovipneumoniae* is determined almost exclusively using nasal swabbing followed by molecular detection with either quantitative PCR or multi-locus sequence typing. However, the diagnostic performance and efficiency of swabbing the nasal passage compared to other anatomical locations has not been determined within sheep populations. The goal of this research was to assess the diagnostic capability of nasopharyngeal swabs in comparison to nasal swabs for the detection of *Mycoplasma ovipneumoniae*.

**Results:**

Nasal and nasopharyngeal swabs were collected during a controlled exposure study of domestic sheep with *Mycoplasma ovipneumoniae*. Both swab types were then analyzed via conventional and quantitative PCR. This dataset showed that the use of nasopharyngeal swabs in lieu of nasal swabs resulted in higher sensitivity, reduced inhibition during quantitative PCR, and higher bacterial copy numbers per swab. Moreover, it was demonstrated that diagnostic sensitivity could be further increased during quantitative PCR via ten-fold dilution of the extracted DNA. To confirm these observations in naturally infected animals, we conducted a field study employing a production flock of domestic sheep using both nasal and nasopharyngeal swabbing techniques. Extracted DNA was assessed using the same molecular techniques, where detection of *Mycoplasma ovipneumoniae* was confirmed by sequencing of either the *rpoB* or 16S rRNA gene. Similar improvements were observed for nasopharyngeal swabs and template treatment methods within the naturally infected flock.

**Conclusions:**

Results demonstrate increased diagnostic sensitivity and specificity when sampling with nasopharyngeal swabs as compared to nasal swabs. Therefore, alternate field-testing strategies employing nasopharyngeal swabs should be considered for diagnosis of the presence of *M. ovipneumoniae*. Importantly, sample treatment following acquisition was found to affect the sensitivity of quantitative PCR, where dilution of eluted DNA template doubled the calculated sensitivity. This demonstrates that, in addition to anatomical location, the presence of inhibitory components in swab extracts also strongly influences diagnostic performance.

**Supplementary Information:**

The online version contains supplementary material available at 10.1186/s12917-024-04342-y.

## Background

Assessing hosts for the presence of pathogens requires sampling the region most applicable to the agent of interest. It is common to isolate bacteria belonging to the Mycoplasma genus from the upper respiratory tract or the genital mucosa of healthy individuals, therefore these microorganisms are considered commensal [1]. However, there are times when overgrowth of Mycoplasmas leads to translocation of the bacterium to other anatomical sites resulting in subclinical or clinical disease [2]. An example of this is *Mycoplasma ovipneumoniae* in domestic sheep, where pneumonic lesions are commonly identified at slaughter in clinically healthy animals [3, 4]. During clinical disease with *M. ovipneumoniae*, domestic sheep tend to present with chronic cough, rectal prolapse, and reduced weight gain [5, 6]. Furthermore, *Mycoplasma ovipneumoniae* has been implicated in spillover events from domestic sheep to wild bighorn sheep, the latter of which tend to suffer more dramatically and result in all-age die offs and poor lamb recruitment for several years following exposure to the pathogen [7, 8]. While both wild and domestic sheep reside in the subfamily Caprinae, the host range for *M. ovipneumoniae* has recently expanded to include the Capreolinae (moose, caribou, mule deer, and white-tailed deer) subfamily [9]. Importantly, isolation of the bacterium from members of the Capreolinae subfamily has occurred in both apparently healthy and clinical animals [9, 10]. Further complicating this situation has been the discovery of a novel Mycoplasma species in moose [11]. Together, these items make it more difficult to tease out the epidemiology of *M. ovipneumoniae.*

Detection of *M. ovipneumoniae* routinely employs nasal swabs, tonsil swabs, and lung tissues for diagnostic testing [9, 12–14]. Unfortunately, the sensitivity and specificity, and associated positive and negative predictive values, of these tests have not been fully considered, where currently available methods have demonstrated inconsistent results [12, 15]. For example, domestic sheep sampling completed within our agency has determined inconsistent bacterial numbers isolated from the nasal cavity and have therefore relied on serial nasal swab sampling to characterize subject shedding [15]. Furthermore, relocation studies of wild sheep suggest poor negative predictive values, where field animals initially test negative but are positive following herd/flock management practices [16, 17]. Within one such study, a bighorn herd initially tested negative for *M. ovipneumoniae* but were subsequently positive and clinically ill following relocation of a subset of the herd [16]. While the authors suggest there was exposure during relocation, the detection of the bacterium in bighorns remaining in the field would indicate that the initial testing may have resulted in false negatives.

The revelation of the expanded host range for *M. ovipneumoniae* [9, 10], the observation that the bacterium can be carried by asymptomatic sheep from both wild and domestic species [17–19], and the discovery of novel species of Mycoplasma [11] reinforce the need for accurate diagnostic strategies with high sensitivity and specificity which can be directly compared between studies. Assessment of domestic sheep infection and carriage of *M. ovipneumoniae* in a controlled study noted that bacterial detections were most consistent when sampling the nasopharynx. Therefore, this study included sampling of a production domestic sheep flock to investigate the effect of using nasopharyngeal swabs in place of nasal swabs to detect *M. ovipneumoniae* in field sheep populations. Testing of sampled swabs employed general detection methods using conventional PCR followed by characterization with quantitative PCR (qPCR) and sequencing of either *rpoB* or 16S rRNA, thereby allowing calculation of test sensitivity and specificity. Thus, the goal of this study is to generate improved diagnostics for the detection of *M. ovipneumoniae*.

## Methods

### Study animals and inoculum

**Fig. 1:**
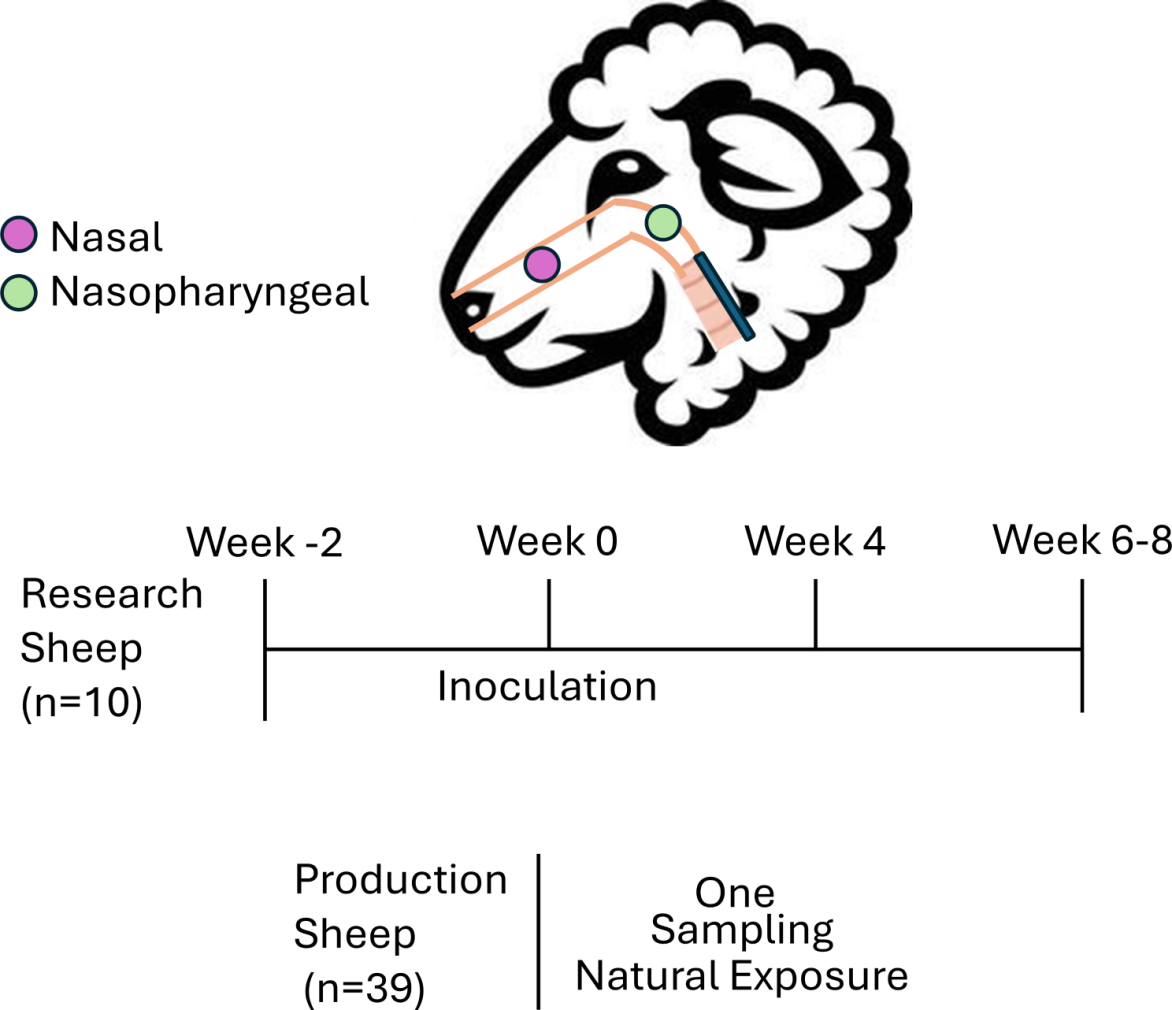
**Diagrammatic representation of swab placement and timing.** Anatomical locations of nasal (purple) and nasopharyngeal (green) swabs. This is followed by the timing for research and production sheep sampling. The animal numbers listed are the total number of sheep present within either group

All animals were maintained under Washington State University’s Institutional Animal Care and Use Committee (IACUC). The sheep described within this study were sourced from two dissimilar settings and management practices, research setting sheep and production setting sheep. Research setting domestic sheep were owned by the USDA-ARS and were approved under animal subject approval form (ASAF) 6870. These sheep were less than a year of age and consisted of six wethers and four ewes. These sheep were free of *M. ovipneumoniae* until subjects were intranasally inoculated with single isolates of the bacterium originating from a domestic sheep flock (Fig. 1). Bacterial inoculum was cultured in an ovine fibroblast (A113) co-culture system that has been described previously [11] with 5*10^6^ copies being instilled within each sheep naris for a total of 1*10^7^ bacteria per sheep.

In contrast, production setting sheep were institutionally owned, raised under production style management, and naturally infected with *M. ovipneumoniae*. The group was comprised of 39 mature ewes (> 1 year old) and were sampled under ASAF 7172.

### Nasal and nasopharyngeal swab collection and DNA extraction

Nasal swabs (BD, BBL CultureSwab) were acquired by entering each naris of the subject with a ventromedial approach while gently twisting the nasal swab. For research setting animals, bronchoalveolar lavage (BAL) was performed on eight of the ten lambs in weeks -2, 4, and 6–8 (relative to inoculation) to assess lower respiratory changes over the experimental time-course (unpublished data). At the time of BAL sampling, the same swab type was used to sample the nasopharynx of each animal via an oral approach during intubation. One lamb was lost during week 4 sampling and is therefore missing endpoint data.

Alternatively, production setting domestic sheep were not sedated and the two swab types (nasal and nasopharyngeal) were collected through the nasal cavity. The nasal swab was taken as described above and was followed by single naris insertion of an 83.82 cm cotton swab for nasopharyngeal sampling. It is estimated that the nasopharyngeal swabs were passed approximately 5 cm further into the nasal cavity as compared to nasal swabs, where the nasopharyngeal distance was estimated using the medial canthus of the eye. Each animal, beyond the two research setting sheep that were not sampled via BAL, had paired nasal and nasopharyngeal swab sampling at indicated timepoints.

Nasal and nasopharyngeal swabs were frozen at -20˚C following sampling. Swab tips were aseptically clipped into a 1.5mL microfuge tube containing 400µL of 1X PBS. DNA was extracted using the Qiagen QIAamp DNA Mini kit with the manufacturer’s protocol, using 2X reagent volumes for lysis and DNA binding. DNA was eluted in 100µL of buffer AE and quantified using the Qubit 4 fluorimeter with the dsDNA broad range quantification kit.

### Conventional and nested PCRs, amplicon sequence verification

Conventional endpoint and nested PCR were run in 20 µl volumes consisting of 10µL Qiagen Multiplex Mastermix, 500nM each of forward and reverse primers, and 2µL of template DNA. Primary screening (herein LM40) was carried out using primers originally described by McAuliffe et al. amplifying a portion of the 16S rRNA from *M. ovipneumoniae* [20]. Cycling conditions were: 95˚C for 15 min followed by 40 cycles of 95˚C for 30 s, 58˚C for 30 s, and 72˚C for 30 s. A final extension step of 72˚C for 10 min was included. Nested PCR of a segment of the *M. ovipneumoniae rpoB* gene [18] was performed when LM40 amplification was either weak, absent, or an inappropriate size. External cycling conditions were: 95˚C for 15 min, 20 cycles of 95˚C 30 s, 58˚C 20 s, and 72˚C two minutes, with a final extension time of 72˚C for five minutes. Internal reaction included 2µL of the resultant external product and cycling conditions were: 95˚C for 15 min, 35 cycles of 95˚C 30 s, 58˚C 30 s, and 72˚C for one minute, with a final extension time of 72˚C for two minutes. Amplicons generated via LM40 and/or nested *rpoB* PCR were verified via Sanger Sequencing (Eurofins Genomics).

### Ovine 439 quantitative PCR

Quantitative PCR (herein ovine 439) targeted a section of a HAD family hydrolase gene of *M. ovipneumoniae* [15] and consisted of 500nM primers 439 F and 439R, 10µL of 2X SsoFast EvaGreen Supermix (Bio-Rad), and 2 µl template DNA in a reaction volume of 20 µl. Nasal and nasopharyngeal DNA were run neat and diluted 1:10 in PCR-grade water. Cycling was performed on either the Bio Rad CFX96 or CFX Opus 96 with the following protocol: 98˚C for two minutes, 40 cycles of 98˚C for five seconds, 57.6˚C for ten seconds, and 72˚C for five seconds. Melt curve analysis was run at 65˚C to 95˚C for five seconds. Samples crossing threshold but failing to cross the melt-curve threshold were considered non-detections.

### Statistical analysis

Version 17.2.0 of JMP software was used to run statistical analyses. Comparison of nonparametric data with two dependent variables employed Wilcoxon exact tests. Statistical measures of DNA quantity (ng/µl) used Excel functions AVERAGE, STDEV, and VAR.S. Calculation of sensitivity, specificity, positive predictive values, and negative predictive values adhered to equations present in [21], where the reference standard relied on sequencing results.

## Results

### Detection of M. Ovipneumoniae in research setting domestic sheep

Research domestic sheep with a minimum of one LM40 detection over the experimental time course are represented in Table 1 (unpublished study). While nasal swabs suggest that 40% of the subjects became infected with *M. ovipneumoniae*, nasopharyngeal swabs detected *M. ovipneumoniae* in 100% of the subjects at week four and 89% of the subjects at week 6–8. Therefore, swabbing the nasopharynx resulted in a 2.5-fold increase in detections within this experiment during week four.

**Table 1:** **LM40 detections ratio from alternate swabs in a research setting**

Week	Nasal Swab	Nasopharyngeal Swab	P-value
-2	0/10	0/8	-
4	4/10	8/8	0.0128*
6–8	1/9	8/9	0.0034**

Timepoint is relative to research animal inoculation. Two animals did not undergo BAL sampling thus no nasopharyngeal swab was taken at week −2 and 4. One animal was lost in week 4. A p-value < 0.05 is indicated with * and < 0.005 is indicated with **

**Fig. 2:**
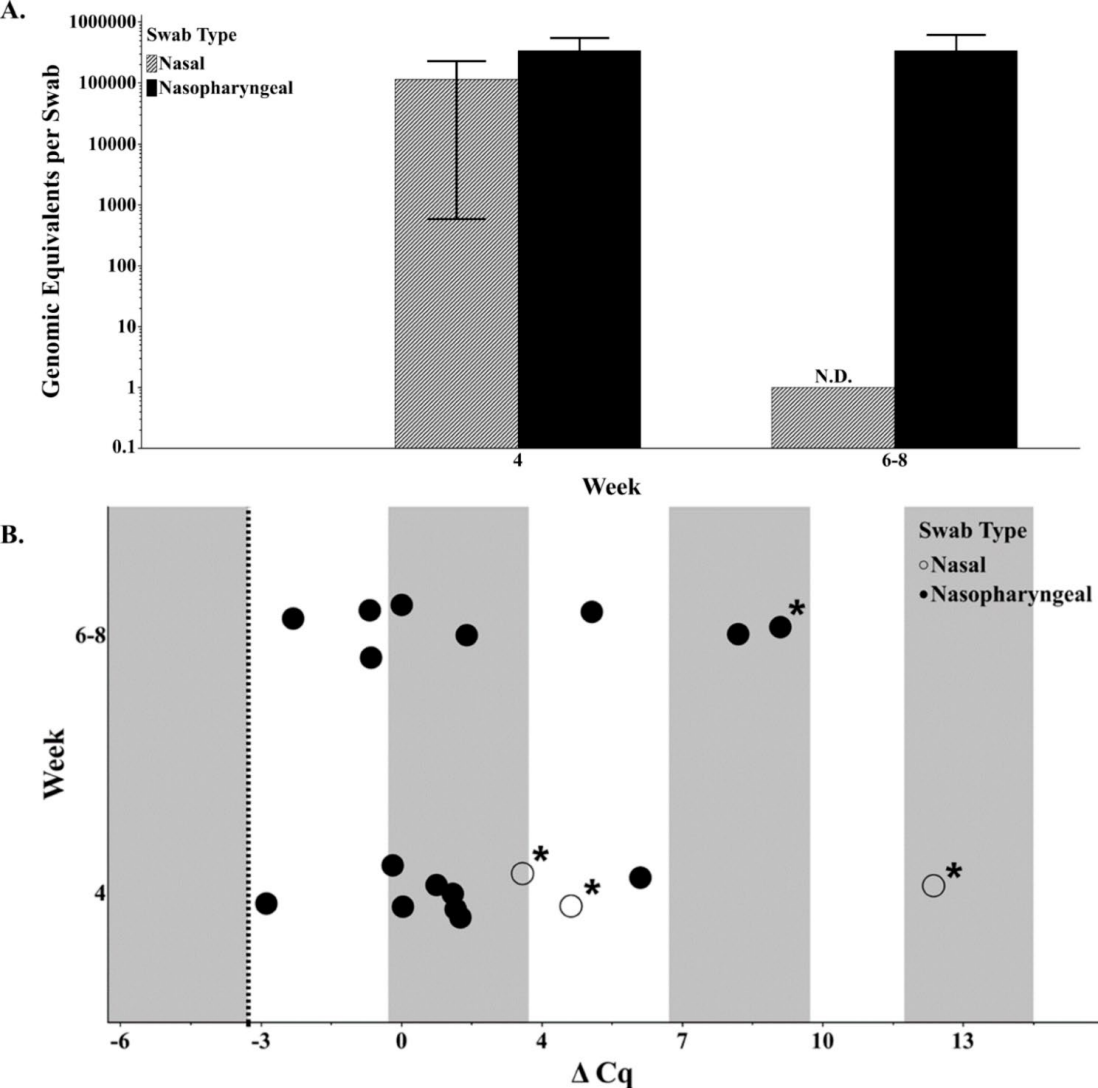
**Nasal versus nasopharyngeal swabs in a research setting. **The week is in relation to inoculation for research domestic sheep. (**A**) Error bars represent standard error of the mean. The nasal swab at week six-eight was a non-detection (ND) on qPCR, where the lowest standard in the curve is 1 bacterial copy. (**B**) The change in cycle threshold (Cq) was calculated by subtracting the 1:10 Cq from the neat Cq. Asterisks indicate a sample that was non-detect during neat sample analysis and had a Cq of 40 substituted. The dashed line at -3.3 indicates an appropriate delta Cq without inhibitors present. Open circles are nasal swabs and filled circles are nasopharyngeal swabs

Swabs that had detection of *M. ovipneumoniae* via conventional LM40 PCR were also assessed via quantitative PCR (qPCR), to determine bacterial load. The resultant qPCR genomic equivalents (GEs) graphed in Fig. 2A allude to higher bacterial copies at weeks 6–8 at the level of the nasopharynx; unfortunately, statistical significance is not met due to the small population size of the research domestic sheep and the single nasal swab tested at this timepoint. Furthermore, qPCR was run on DNA at both neat and 1:10 dilutions. This allows for the assessment of inhibitors, where the standard curve suggests that a tenfold dilution is equivalent to a -3.3 change in cycle threshold (Supplemental Fig. 1). Figure 2B shows that both nasal and nasopharyngeal samples are inhibited within qPCR, but that the level of inhibition was generally less for nasopharyngeal swabs than that of nasal swabs. This is further evidenced by the observation that three nasal swabs at week four were non-detections in qPCR until a 1:10 dilution of the sample was performed.

### Detection of M. ovipneumoniae in production setting domestic sheep

**Fig. 3:**
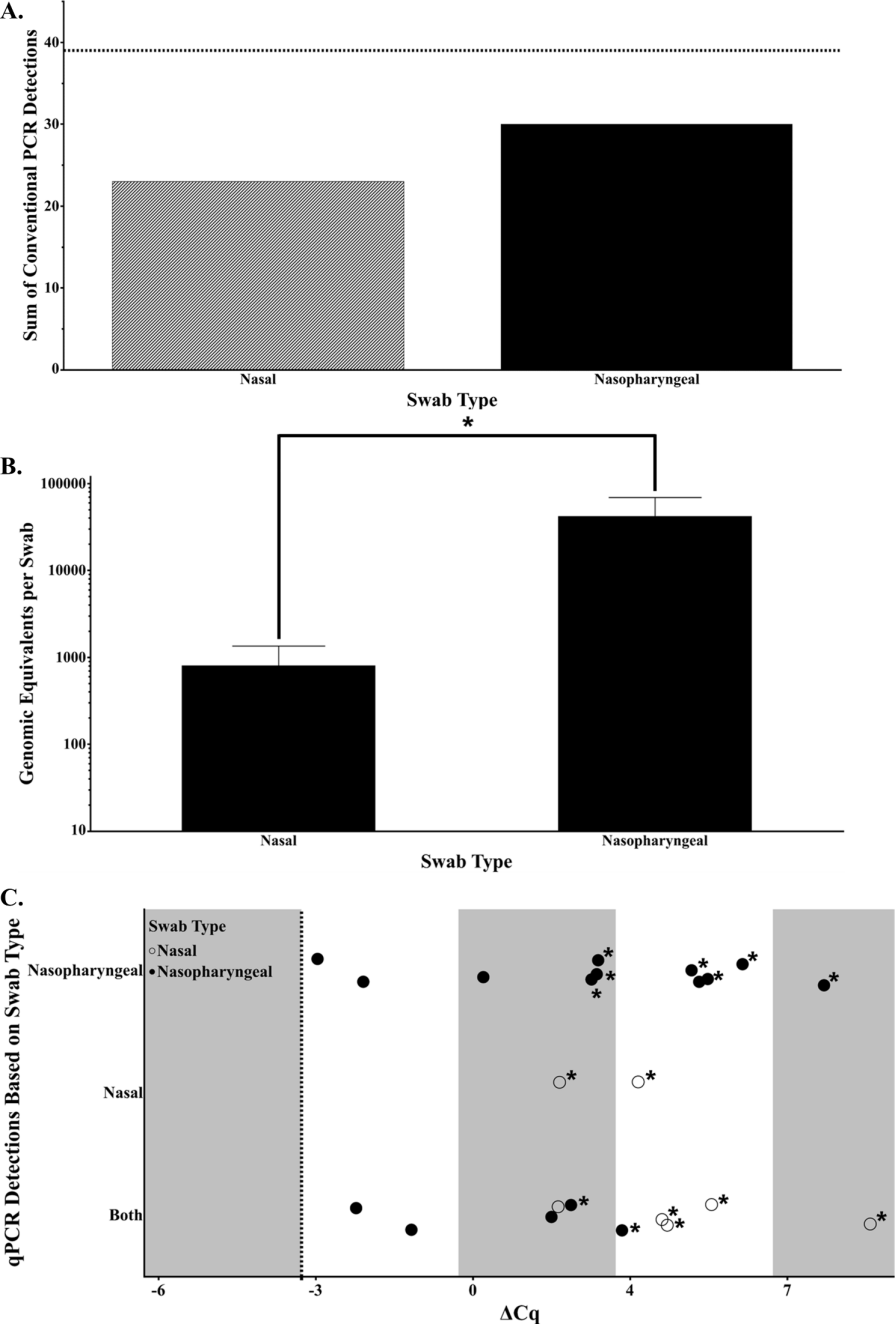
**Nasal versus nasopharyngeal swabs in a production setting.** (**A**) Conventional LM40 PCR detections summed for nasal (diagonal bars) and nasopharyngeal (solid black bars) swabs. The dashed line is the total number of animals tested. (**B**) Error bars represent standard error of the mean, where the type of swab graphed is on the x-axis. An * indicates a p-value < 0.05. (**C**). The change in Cq was calculated as done in Fig. 2B, asterisks indicate a non-detect of a sample when run neat. The dashed line at -3.3 indicates an appropriate delta Cq for a tenfold dilution. Detections were categorized as nasopharyngeal swab only, nasal swab only, or both swab types. Open circles are nasal swabs and filled circles are nasopharyngeal swabs

The observed increase in PCR detections when using nasopharyngeal swabs in a research setting prompted us to mimic the experiment in a production setting. Using the molecular techniques described above, we demonstrate a 17.9% increase in detections with nasopharyngeal swabs (Fig. 3A), trending towards significance with a p-value of 0.1554. In contrast to the research setting sheep, production animals carried significantly more bacterial GEs when swabbing the nasopharynx as compared to the nasal cavity (Fig. 3B). A difference in the presence of qPCR inhibitors between the two swab types was not expected due to both sampling techniques entering through the nasal cavity within the production setting. However, when assessing the effects of dilution on the resultant Cq values, nasopharyngeal swabs were more likely to be near the appropriate delta Cq (Fig. 3C). Furthermore, use of diluted DNA resulted in a percent increase in *M. ovipneumoniae* detections in 85.7% of nasal swabs but only 56.3% of nasopharyngeal swabs.

### Contamination of swab types with host or environmental DNA

**Fig. 4:**
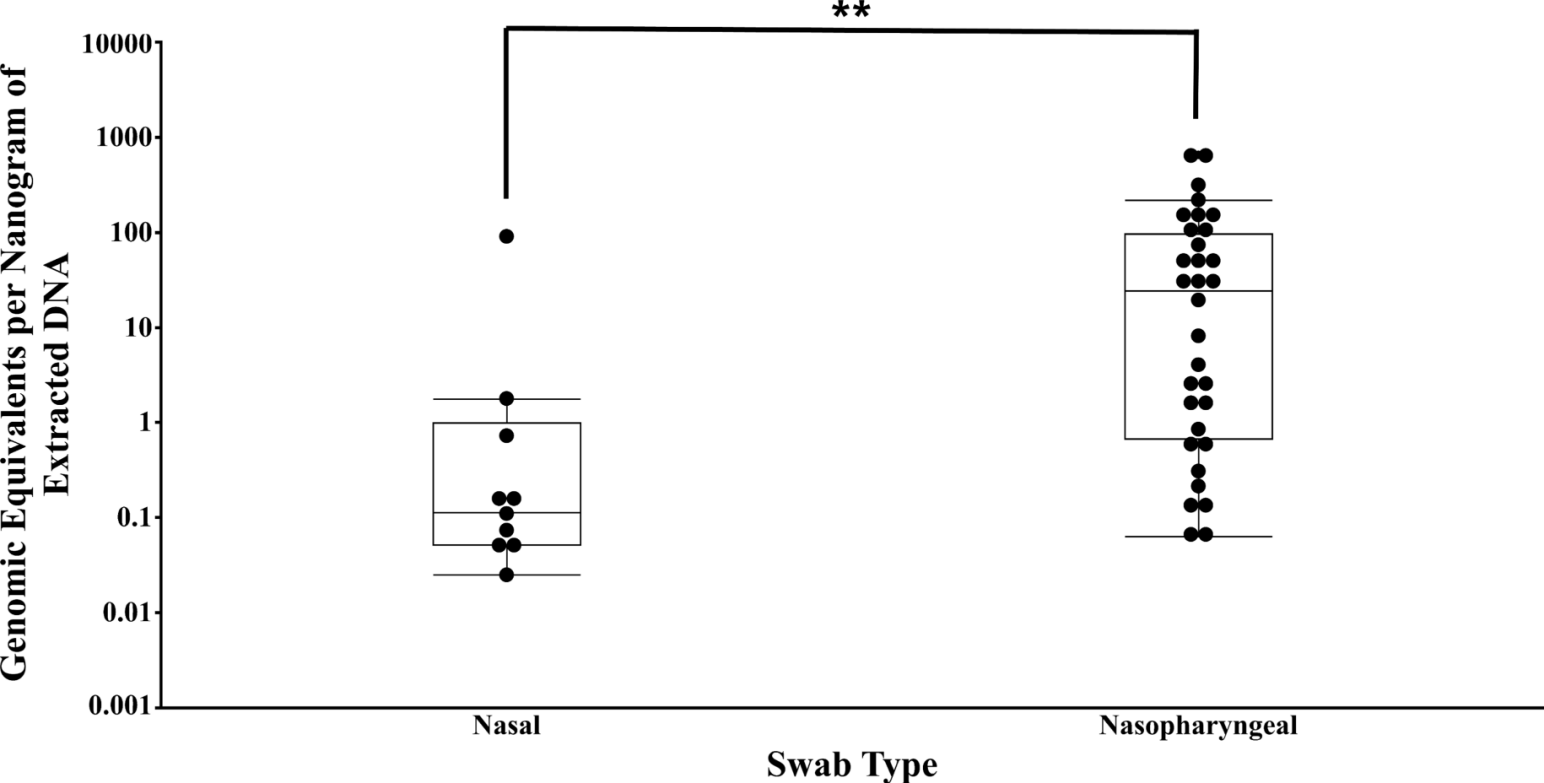
**Bacterial copy number per nanogram of extracted DNA based on swab type.** Each filled circle represents a single sample within the box plot. A statistical Wilcoxon exact test was run, and the resultant p-value was < 0.005 (**)

Swabbing of the nasal cavity tends to result in variable amounts of mucus, debris, and host cells. For this reason, the amount of DNA extracted from nasal and nasopharyngeal swabs was measured and compared (Table 2). DNA extracted from nasal swabs exhibits higher sample variation in DNA quantity compared to nasopharyngeal swabs. It is assumed that this is due to swab contamination with host or environmental DNA. Contamination is suspected when PCR amplicons are inappropriately sized and/or when inconsistent amplification between LM40 and nested *rpoB* is observed. Further evaluation of the level of host contamination of swabs was calculated by dividing the GEs per nanogram of extracted DNA for all research and production swabs run in qPCR (Fig. 4). Nasopharyngeal swabs had significantly higher copy numbers of *M. ovipneumoniae* per amount of extracted DNA as compared to nasal swabs.

**Table 2:** **Statistical distributions of extracted nasal and nasopharyngeal swab DNA**

	Research Setting		Production Setting	
	Nasal Swabs	Nasopharyngeal Swabs	Nasal Swabs	Nasopharyngeal Swabs
Average	122.8 ng/µl	16.3 ng/µl	93.7ng/µl	26.5ng/µl
SD	96.0	18.6	43.6	14
Range	23.1–340 ng/µl	0.9–84.8 ng/µl	32.4–200 ng/µl	0.43–62.4 ng/µl
SV	9169.7	344.3	1853.2	191.1

The average, standard deviation (SD), and sample variance (SV) were calculated from 29 nasal swabs and 25 nasopharyngeal swabs from research setting subjects and 39 nasal and nasopharyngeal swabs from production setting subjects

### Statistical assessment of the current molecular diagnostics

Within this study, we calculated nasal and nasopharyngeal swab sensitivity, specificity, positive predictive values (PPV), and negative predictive values (NPV) using amplicon sequencing as the diagnostic test reference standard (Table 3). The specificity of conventional LM40 PCR drastically differs between the research (91.6–100%) and production (11.1%) setting animals. Importantly, research setting subjects were negative for *Mycoplasma* species prior to inoculation (Week −2, Table 1) while production setting subjects also carried a *Mycoplasma conjunctivae-*like species (~ 94% identical at the LM locus), increasing the test’s false positive rate [11]. In contrast, the specificity of the qPCR was 100% in either the research or production setting, failing to detect the alternate *Mycoplasma* species identified in the production setting. The sensitivity of the ovine 439 qPCR is drastically reduced when testing undiluted DNA, where some recovery in sensitivity occurs when templates are diluted 1:10 (Table 3). Notably, nasopharyngeal swabs have a percent sensitivity that is 2.6-fold higher and a negative predictive value percentage that is 1.6-fold higher than nasal swabs.

**Table 3:** **Diagnostic test statistics for all molecular tests and animal care settings**

	Research Setting		Production Setting	
	Nasal Swabs	Nasopharyngeal Swabs	Nasal Swabs	Nasopharyngeal Swabs
**LM40 Conventional PCR**				
Sensitivity	29.4%	94.1%	72.7%	100%
Specificity	92.9%	100%	11.1%	11.1%
PPV	83.3%	100%	66.7%	73.3%
NPV	52%	93.3%	14.3%	100%
**Ovine 439 qPCR (Neat)**				
Sensitivity	0%	88.2%	4.5%	31.8%
Specificity	100%	100%	100%	100%
PPV	0%	100%	100%	100%
NPV	45.2%	85.7%	30%	37.5%
**Ovine 439 qPCR (1:10)**				
Sensitivity	17.6%	94.1%	27.3%	72.7%
Specificity	100%	100%	100%	100%
PPV	100%	100%	100%	100%
NPV	50%	92.3%	36%	60%

A combination of molecular techniques (conventional PCR, quantitative PCR, and sequencing of PCR amplicons) was employed to determine if a sample contained *M. ovipneumoniae*. From these results, the sensitivity, specificity, positive predictive value (PPV), and negative predictive value (NPV) were calculated for each sampling and molecular technique employed [21]

## Discussion

The data presented herein demonstrate the improvements that modifications to current diagnostic methods for detection and quantification of *M. ovipneumoniae* would yield. Alteration of the anatomical sampling site to the nasopharynx increased the sensitivity, negative predictive value, and qPCR dynamic detection ranges as compared to rostral nasal cavity swabbing (Fig. 4; Table 3). Nasopharyngeal sampling was employed following the observation that sedation and intubation of research setting subjects affected the ability to detect *M. ovipneumoniae* when using nasal swabs. Repeated sampling of the nasopharynx resulted in significantly more detections of the bacterium over the course of our inoculation study (Table 1), supporting continued research of this anatomical site. In contrast, production setting animals were not sedated, and while this likely decreased the difference in overall detections between swab types (Fig. 3A), there were still significantly more genomic copies detected at the level of the nasopharynx (Fig. 3B). It is therefore suggested that the nasopharynx is the proper site of replication of *M. ovipneumoniae* within this species. A prior study examining the microbiome of the nasal cavity within cattle supports the *Mycoplasma* replicative niche at the level of the nasopharynx as the *Mycoplasmatales* Order represented approximately one-third of the DNA on a nasal swab but two-thirds of the DNA present on a nasopharyngeal swab [22].

At present, the field defines chronic carriers as hosts which have nasal swab detection of *M. ovipneumoniae* two or more times [13, 19]. However, the difference in the detections observed between nasal and nasopharyngeal swabs in Table 1 advocates for a more precise definition between a carrier and shedder. We would recommend that subjects be considered chronic shedders when serial nasal swab tests are employed to detect *M. ovipneumoniae*, while definition of a chronic carrier would utilize nasopharyngeal swabs. As depicted in Table 1 inoculated research setting subjects had nasal swab detection at week four that generally ceased by weeks six to eight. In contrast, use of nasopharyngeal swabs resulted in continued detection of the bacterium, indicating these subjects were carriers that were no longer shedding a detectable level of the bacterium via nasal swabs. It is therefore conceivable that the distinction between nasal shedders and nasopharyngeal carriers prove to be important in the transmissibility of the bacterium.

Additional benefits of this work show the effect that downstream testing and processing can have on diagnostic results. While conventional PCR is routinely employed as a screening test, it is typically followed by additional characterization using qPCR and/or strain typing via multi-locus sequence typing (MLST) [16, 23, 24]. The conventional primers (LM40) designed by McAuliffe et al. are prominently featured within *M. ovipneumoniae* diagnostic testing and are suggested to have a high specificity, recognizing only *M. bovoculi* from alternatively tested Mycoplasmas, and sensitivity, detecting as low as 10 copies of the bacterial genome [25]. Accordingly, we have found the LM40 conventional PCR to have a higher sensitivity as compared to ovine 439 qPCR (Table 3). While it was previously demonstrated that American isolates of *M. ovipneumoniae* lacked the HindIII digestion site that distinguished between *M. bovoculi* and *M. ovipneumoniae* species during conventional LM40 [26], the present study describes amplification of a *M. conjunctivae*-like species. Accordingly, the specificity of this test is diminished, and could be further reduced as alternate strains or species of Mycoplasmas are identified. It is therefore imperative that conventional PCR detections be followed up with a secondary methodology, as is the current gold standard of conventional PCR followed by sequencing (direct communication with Washington Animal Disease Diagnostic Lab, WADDL).

Furthermore, our laboratory has observed that the quantitation of bacterial load via qPCR using DNA extracted from nasal swabs is frequently inaccurate due to the presence of inhibitors [27]. While the use of nasopharyngeal swabs decreased the level of inhibition (Figs. 2B and 3C), we were not able to completely resolve the issue using this alternate swabbing technique. Therefore, extracted DNA, from either swab type, was assessed both neat and using a 1:10 dilution (Table 3). This resulted in an increase of test sensitivity and negative predictive value regardless of the anatomical location tested. Two other fee-for-service diagnostics using qPCR are available, one from Kansas State University [25] and the second from WADDL [28, 29], both of which utilize detection of the 16S rRNA gene. The test at Kansas State University offers a multiplex design to differentiate *M. ovipneumoniae* from the *M. conjunctivae-*like species [25]; however it is important to remember that the Mycoplasmatales represents an ever-expanding Order of bacterium, where *Mycoplasma bovis* and *Mycoplasma arginini* represent two other common species which may be isolated from the upper respiratory tract of ungulate animals [30]. The researchers therefore maintain that it is valuable to perform sequence verification as a final step to ensure the species of *Mycoplasma* is correctly identified.

While the present study sampled domestic sheep species, it would be imperative to determine if nasopharyngeal swabbing impacts the results seen during sampling of bighorn sheep species. In 2017, Butler et al. calculated that if the prevalence of *M. ovipneumoniae* within a bighorn herd was 0.1, then the herd could be diagnosed as having carriers following testing of approximately 20 animals [12]. If the present dataset holds true in bighorn sheep, then fewer sheep would require sampling when employing nasopharyngeal swabs. Notably, many bighorn sampling events require the use of chemical restraint to obtain nasal swabs and therefore would not require an extraneous amount of additional effort to also gather nasopharyngeal swabs [12, 16, 23]. The significance of sampling and sedating fewer bighorns is the suggested effect to bighorn recruitment, where recent studies assessing post-capture mortality and capture-induced abandonment propose a negative effect on the recovery of bighorn sheep populations [24].

It would be of significant value to have consistent diagnostic testing methods within the *M. ovipneumoniae* field. The outcome of which would aid in increased comparison between both study and host types. Overall, this work suggests that nasopharyngeal sampling is a more accurate and reliable measure of *M. ovipneumoniae* prevalence in domestic sheep herds. Importantly, this reliability was further increased during qPCR reactions when the effect of inhibitors was ameliorated. Presently, this was accomplished by dilution of the template DNA, which may coincidentally reduce the test sensitivity. The authors therefore suggest additional research be performed using alternate extraction methods in conjunction with the discussed sampling and molecular diagnostic tests.

## Electronic supplementary material

Below is the link to the electronic supplementary material.

Supplementary Material 1

## Data Availability

The datasets used and/or analyzed during the current study are available from the corresponding author on reasonable request.
