# Supplementary material for: Upper respiratory tract detection of Mycoplasma ovipneumoniae employing nasopharyngeal swabs
Source: BMC Vet Res. 2024 Nov 1;20:502. doi: 10.1186/s12917-024-04342-y (PMC11529185; doi:10.1186/s12917-024-04342-y)
Supplement: Supplementary file 1 — Supplementary Material 1 [file 12917_2024_4342_MOESM1_ESM.docx]

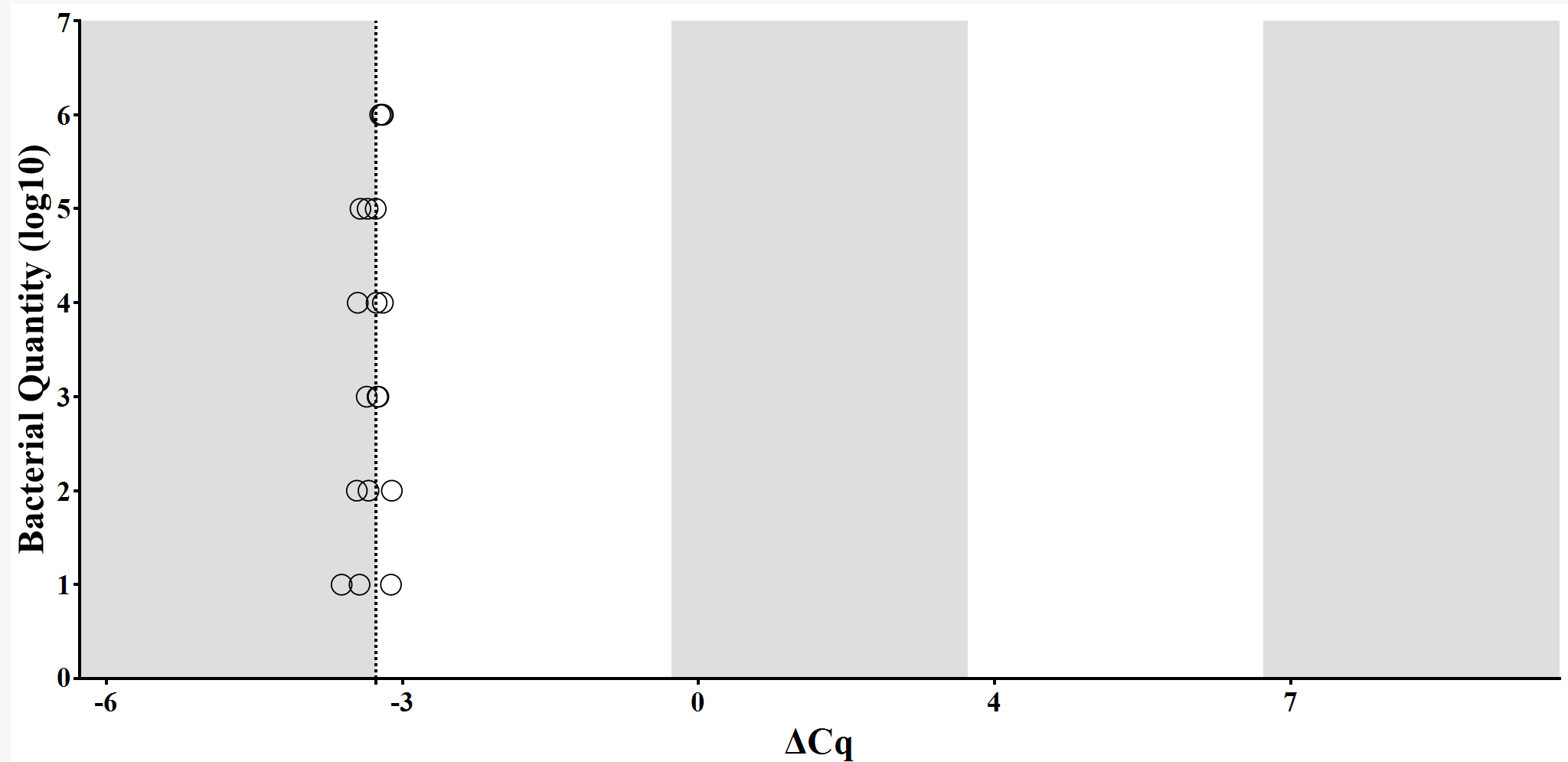

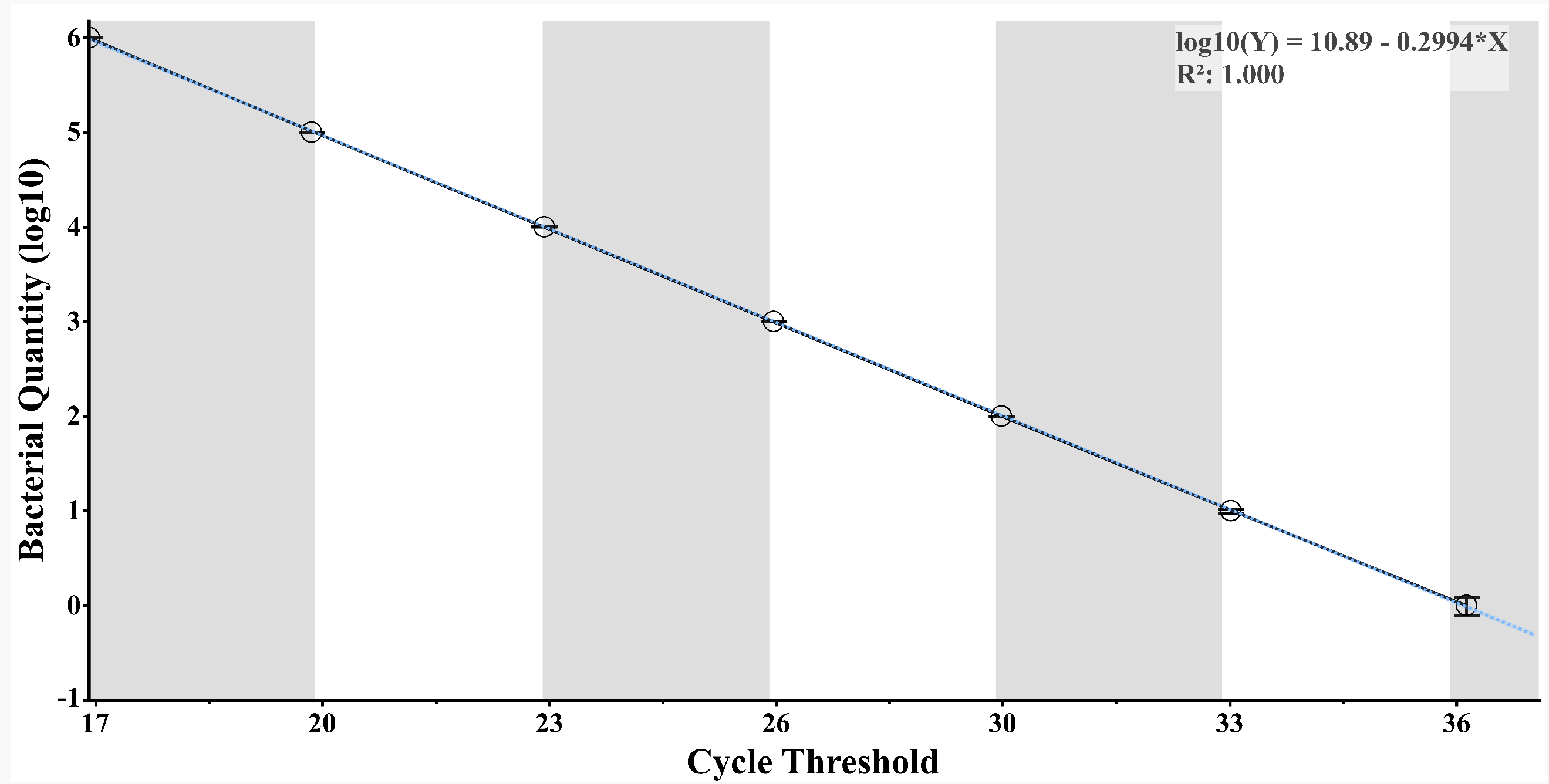


**B.**

**A.**

**Supplemental Figure 1: Standard curve for Ovine 439 qPCR.**

DNA for the standard curve is isolated from Type Strain Y98 *M. ovipneumoniae*. The data represents three independent qPCR experiments, where the reaction efficiencies were 100.5, 98.5, and 98.9 percent. (**A**) Bacterial quantity is plotted on the y-axis and cycle Cq is plotted on the x-axis. Standard deviations are graphed for each bacterial quantity in the standard curve. (**B**) The cycle threshold (Cq) for the smaller bacterial quantity was subtracted from the Cq of the larger bacterial quantity for each of the three standard curve reactions. The gray versus white vertical bars from the x-axis indicate a 3.3 change in cycle threshold integer.
